# Cost-Effective Calculation of Collective Electronic Excitations in Graphite Intercalated Compounds

**DOI:** 10.3390/nano12101746

**Published:** 2022-05-20

**Authors:** Pengfei Suo, Li Mao, Jing Shi, Hongxing Xu

**Affiliations:** 1 School of Physics and Technology, Wuhan University, Wuhan 430072, China; pfsuo@whu.edu.cn; 2 Wuhan Institute of Quantum Technology, Wuhan 430072, China; 3 School of Microelectronics, Wuhan University, Wuhan 430072, China

**Keywords:** intercalation, plasmon, tight-binding model, local-field effects

## Abstract

Graphite/graphene intercalation compounds with good and improving electrical transport properties, optical properties, magnetic properties and even superconductivity are widely used in battery, capacitors and so on. Computational simulation helps with predicting important properties and exploring unknown functions, while it is restricted by limited computing resources and insufficient precision. Here, we present a cost-effective study on graphite/graphene intercalation compounds properties with sufficient precision. The calculation of electronic collective excitations in AA-stacking graphite based on the tight-binding model within the random phase approximation framework agrees quite well with previous experimental and calculation work, such as effects of doping level, interlayer distance, and interlayer hopping on 2D π plasmon and 3D intraband plasmon modes. This cost-effective simulation method can be extended to other intercalation compounds with unlimited intercalation species.

## 1. Introduction

The physical properties can be modified by intercalation significantly in graphite or graphene intercalated compounds (GICs) [1,2], such as electrical transport properties [3], optical properties [4], magnetic properties [5] and even superconductivity [6,7,8,9,10]. Superconductivity in the alkali GICs was first reported in 1965 [6], with critical temperature below 1 K. After the critical temperature rising to 11.5 K of CaC${}_{6}$ was reported [7], great interest has arisen for electronic properties of GICs [11,12,13,14,15,16].

The electronic collective excitations play a significant role in optical and dielectric properties, which have been studied both in experiments [17,18,19,20] and in calculations [21,22,23,24,25,26,27] in GICs. The low-energy π plasmon mode, which exists in pure graphite at 7 eV in optical limit, was found to shift to lower energy in GICs [17,18,19,20,22,23,24]. In addition, an intraband plasmon (IP) mode appeared with energy ∼1 eV in GICs due to the doping effects [18,19,20,21,23,24]. Furthermore, acoustic plasmon (AP) may exist and could play a role in superconductivity [20,23,24].

Theoretically, the plasmon properties are studied in a tight-binding (TB) model and time-dependent density functional theory (TDDFT). Shung [21] reported that the in-plane IP mode can be well described in a layered 2D two-band TB model near the Dirac cone, where the interlayer tunneling effects are neglected and only Coulomb interaction in different layers is retained. Lin et al. [22] revealed the in-plane π plasmon properties can also be depicted in this model, where the bandstructure in the whole first Brillouin zone (1BZ) has to be taken into account. Echeverry et al. [23,24] studied the dielectric properties of GICs at energies below 12 eV by the state-of-the-art first-principle calculations. Bulk plasmon (BP), π plasmon, IP and AP modes are discussed in LiC${}_{6}$, CaC${}_{6}$, SrC${}_{6}$, and BaC${}_{6}$, which is in good agreement with the experimental results. However, the first-principle calculation is too time-consuming, and when the species of intercalation changes, the band structure, interlayer distance, and doping level are changed simultaneously, and it is hard to distingulish their effects on plasmons.

Computational simulation is a green tool for new materials developing and property prediction which could be used in many research studies. However, limited computing resources and insufficient precision restricted the promotion of wide usage. In the present work, in order to understand how the in-plane and out-plane electronic collective excitations are affected by different parameters in GICs, we extend the two-band TB model of AA-stacking graphite, where the interlayer tunneling effects are also taken into consideration. In this model, we study the low-energy electronic collective excitations within the random phase approximation (RPA) framework. The local field effects (LFE) are also involved. Our calculations show that doping level and interlayer hopping affect plasmons mainly by band structure effects, such as density of states (DOS) and group velocity near the Fermi level, and the forbidden effect of interband transition, while interlayer distance modifies plasmons via the long-range interlayer Coulomb correlations. In addition, stacking order can modify interlayer coupling and have a great influence on plasmon properties.

## 2. Method

For periodic systems, the dielectric function can be written in terms of tight-binding basis in the form

(1) $$\begin{array}{c} \epsilon_{G,G^{\prime }}\left(q,\omega \right)=\delta_{G,G^{\prime }}-v\left(q+G\right)\chi_{G,G^{\prime }}^{0}\left(q,\omega \right), \end{array}$$

where **G**,**G**’ are reciprocal-lattice vectors, **q** is a wave vector restricted to the first Brillouin zone (1BZ), and $v\left(q\right)=4\pi e^{2}/\left(\Omega q^{2}\right)$ is the Fourier transform Coulomb potential, Ω being the volume of the unit cell. The polarizability function $\chi^{0}$ is expressed as [28]

(2) $$\begin{array}{c} \chi_{GG^{\prime }}^{0}\left(q,\omega \right)=\sum_{ss^{\prime }}A_{s}\left(q,G\right)N_{ss^{\prime }}\left(q,\omega \right)A_{s^{\prime }}^{*}\left(q,G^{\prime }\right), \end{array}$$

where

(3) $$\begin{array}{cc} N_{ss^{\prime }}\left(q,\omega \right) & =\frac{2}{N}\sum_{nn^{\prime }k}\frac{f_{nk}-f_{n^{\prime }k+q}}{\hbar \omega +E_{nk}-E_{n^{\prime }k+q}+i\hbar \eta }h\left(k\right)\\ \times & C_{\nu }^{*}\left(nk\right)C_{\mu }\left(n^{\prime }k+q\right)C_{\mu^{\prime }}^{*}\left(n^{\prime }k+q\right)C_{\nu^{\prime }}\left(nk\right), \end{array}$$

$E_{nk}$ and $f_{nk}$ are the eigen-energy and Fermi–Dirac occupuation for band index *n* and wave vector **k**, $C_{\mu }\left(nk\right)$ is the contribution of the μth tight-binding basis function to the Hamiltonian eigenstate (for more details, please see Appendix A), *N* is the number of unit cells, η is a broadening parameter, and $h\left(k\right)=\exp[ik\cdot \left(R_{L}-R_{L^{\prime }}+\tau_{\mu }-\tau_{\nu }+\tau_{\mu^{\prime }}-\tau_{\nu^{\prime }}\right)]$ is a phase factor. Factor 2 accounts for spin (we assume a spin-degenerate system).

(4) $$\begin{array}{c} A_{s}\left(q,G\right)=\langle \nu ,0|e^{-i(q+G)\cdot r}e^{iq\cdot \tau_{\mu }}|\mu ,L\rangle \end{array}$$

is defined as the charge-density wave. The index *s* stands for the lattice vector index *L* and for the indices ν and μ of the orbital. Using these relations, the dielectric matrix can be written in the form

(5) $$\begin{array}{cc} \epsilon_{GG^{\prime }}\left(q,\omega \right)= & \delta_{GG^{\prime }}-v\left(q+G\right)\\ \; & \times \sum_{ss^{\prime }}A_{s}\left(q,G\right)N_{ss^{\prime }}\left(q,\omega \right)A_{s^{\prime }}^{*}\left(q,G^{\prime }\right). \end{array}$$

The separable form of the susceptibility matrix in Equation (5) enables us to calculate the inverse dielectric matrix [29]

(6) $$\begin{array}{cc} \epsilon_{GG^{\prime }}^{-1}\left(q,\omega \right)= & \delta_{GG^{\prime }}+v\left(q+G\right)\\ \; & \times \sum_{ss^{\prime }}A_{s}\left(q,G\right)S_{ss^{\prime }}\left(q,\omega \right)A_{s^{\prime }}^{*}\left(q,G^{\prime }\right), \end{array}$$

where

(7) $$\begin{array}{c} S_{ss^{\prime }}\left(q,\omega \right)=\sum_{s_{1}}N_{ss_{1}}\left(q,\omega \right)T_{s_{1}s^{\prime }}^{-1}, \end{array}$$

and

(8) $$\begin{array}{c} T_{\alpha \alpha^{\prime }}\left(q,\omega \right)=\delta_{\alpha \alpha^{\prime }}-\sum_{\alpha_{1}}V_{\alpha \alpha_{1}}\left(q\right)N_{\alpha_{1}\alpha^{\prime }}\left(q,\omega \right), \end{array}$$

where the Coulomb interaction between the charge-density waves is [29]

(9) $$\begin{array}{cc} V_{\alpha \alpha_{1}}\left(q\right) & =\sum_{G^{\prime \prime }}A_{\alpha }^{*}\left(q,G^{\prime \prime }\right)v\left(q,G^{\prime \prime }\right)A_{\alpha_{1}}\left(q,G^{\prime \prime }\right)\\ \; & =\sum_{m}e^{-iq\cdot (R_{m}+\tau_{\mu }-\tau_{\mu^{\prime }})}\int dr\int dr^{\prime }\phi_{\mu }^{*}\left(r-R_{L}-R_{m}\right)\\ \; & \times \phi_{\nu }\left(r-R_{m}\right)v\left(r-r^{\prime }\right)\phi_{\nu^{\prime }}^{*}\left(r^{\prime }\right)\phi_{\mu^{\prime }}\left(r^{\prime }-R_{L^{\prime }}\right). \end{array}$$

The off-diagonal elements of the $\chi_{GG^{\prime }}^{0}$ matrix describes the response of the electrons at wave vectors different from the external perturbing field and thus contain information about the inhomogeneity of the microscopic response of electrons known as the local field effect (LFE) [30]. The macroscopic dielectric function is defined as

(10) $$\begin{array}{c} \epsilon_{M}\left(q,\omega \right)=\frac{1}{\epsilon_{00}^{-1}\left(q,\omega \right)}, \end{array}$$

If we neglect the LFE, it becomes $\epsilon_{M}\left(q,\omega \right)=\epsilon_{00}\left(q,\omega \right)$. This macroscopic dielectric function is directly related to many experimental properties. For example, the optical-absorption spectrum (ABS) is given by Im$\epsilon_{M}\left(q\to 0,\omega \right)$. The electron energy-loss spetrum (EELS) is proportional to −Im(1/ϵM). EELS is especially useful in probing the collective electronic collective excitations, known as plasmons, of bulk and low-dimensional systems.

Without loss of accuracy, we make some approximations to avoid calculating the integral of tight-binding basis function, as shown in Appendix B, to accelerate calculation greatly.

## 3. Results and Discussion

### 3.1. TB Model of AA-Stacking Graphite

**Figure 1:**
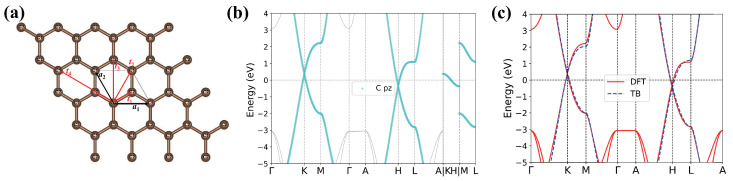
Lattice and electronic structure of AA-graphite. (**a**) Top view of atomic structure of AA-stacking graphite. The primitive cell, lattice vectors **a**${}_{1}$, **a**${}_{2}$ and in-plane hopping **t** are indicated. (**b**) Band structure of AA-stacking graphite, with the corresponding $p_{z}$ orbitals in cyan. The size of the symbol represents the orbital weight. (**c**) Comparison of the band structure for AA-stacking graphite obtained from DFT (red line) and TB (blue dashed line) calculations, respectively.

In AA-stacking graphite, layers of carbon atoms locate directly on top of each other, as shown in Figure 1a. In order to obtain a reliable TB model, the geometrical optimization and electronic properties are performed by first-principle calculations (for more details, please see Appendix C). The calculated band structure in the vicinity of the Fermi level along the high-symmetry directions of 1BZ is shown in Figure 1b, which is in good agreement with the previous results [31]. The bonding π and antibonding $\pi^{*}$ bands dominate the band structure. A hole pocket appears at K and an electron pocket appears at H.

After fitting the TB parameters, we constructed TB Hamiltonian as

(11) $$\begin{array}{c} H\left(k\right)=\left(\begin{array}{cc} H_{11}\left(k\right) & H_{12}\left(k\right)\\ H_{21}\left(k\right) & H_{22}\left(k\right) \end{array}\right) \end{array}$$

where

(12) $$\begin{array}{cc} H_{21}\left(k\right)= & H_{12}^{*}\left(k\right),\\ H_{22}\left(k\right)= & H_{11}\left(k\right),\\ H_{11}\left(k\right)= & \epsilon_{p}+t_{2}g_{2}\left(k\right)+t_{4}g_{4}\left(k\right)+t_{⊥}g_{⊥}\left(k\right),\\ H_{12}\left(k\right)= & t_{1}g_{1}\left(k\right)+t_{3}g_{3}\left(k\right), \end{array}$$

$\epsilon_{p}$ is the on-site energy of $p_{z}$ orbital of C atoms (0.51 eV in this work), and

(13) $$\begin{array}{cc} g_{1}\left(k\right)= & e^{ik\cdot (\frac{2}{3}a_{1}+\frac{1}{3}a_{2})}+e^{ik\cdot (-\frac{1}{3}a_{1}+\frac{1}{3}a_{2})}+e^{ik\cdot (-\frac{1}{3}a_{1}-\frac{2}{3}a_{2})},\\ g_{2}\left(k\right)= & 2\cos\left(k\cdot a_{1}\right)+2\cos\left(k\cdot a_{2}\right)+2\cos\left(k\cdot \left(a_{1}+a_{2}\right)\right),\\ g_{3}\left(k\right)= & e^{ik\cdot (\frac{2}{3}a_{1}+\frac{4}{3}a_{2})}+e^{ik\cdot (\frac{2}{3}a_{1}-\frac{2}{3}a_{2})}+e^{ik\cdot (-\frac{4}{3}a_{1}-\frac{2}{3}a_{2})},\\ g_{4}\left(k\right)= & 2\cos\left(k\cdot \left(2a_{1}+a_{2}\right)\right)+2\cos\left(k\cdot \left(a_{1}+2a_{2}\right)\right)\\ \; & +2\cos(k\cdot \left(-a_{1}+a_{2}\right)),\\ g_{⊥}\left(k\right)= & 2\cos(k\cdot a_{⊥}). \end{array}$$

The detail values of hopping parameters, schematically shown in Figure 1a, are listed in Table 1. From this TB Hamiltonian, energy eigenvalues at high-symmetry points can be calculated analytically (listed in Table 2). The band structures derived from our TB model match well with DFT calculation (Figure 1c), implying a reliable TB model.

### 3.2. Plasmon Excitations

**Figure 2:**
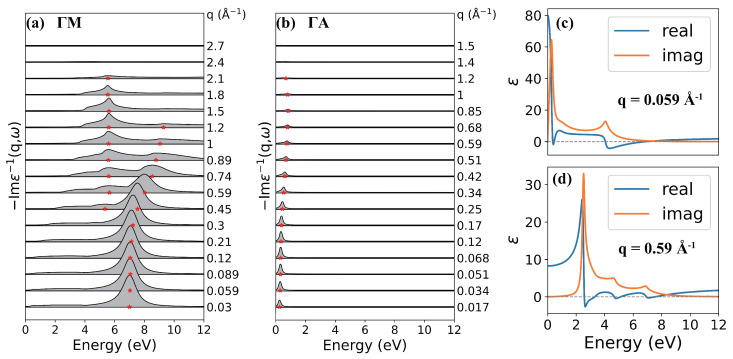
Calculated loss functions along (**a**) the ΓM and (**b**) ΓA directions as a function of *q*. The peak positions are marked by red stars. Real and imaginary part of dielectric function at (**c**) q=0.059 Å and (**d**) q=0.59 Å along the ΓM direction.

By the method introduced in Section 2, we calculated the excitation spectra of AA-stacking graphite with and without inclusion of the LFE. T=0 K, η=0.1 eV, $\epsilon_{0}=2.4$ [32] (more details in Appendix B) and a dense 240×240×180 **k**-mesh were used in all calculations. In Figure 2, the loss function is shown as a function of energy at variable momentum transfer *q* along ΓM (a) and ΓA (b) directions.

In the ΓM (in-plane) direction, the main feature of the loss spectra is a strong high-energy peak around 7 eV at small *q*. As *q* increases, it shows a parabolic-like positive dispersion and splits into two peaks at large momentum (q>0.3 Å${}^{-1}$). This peak is attributed to the collective interband transitions from π to $\pi^{*}$ band around M point and it is assigned as π plasmon [33]. The split features of π plasmon have also been reported in monolayer graphene [34]. With the help of real and imaginary parts of the dielectric function at q=0.059 Å${}^{-1}$ (Figure 2c) and q=0.59 Å${}^{-1}$ (Figure 2d), it is clear that the peaks splitting at large *q* originates from the splitted collective interband transitions. In addition, there exists a low-energy intraband plasmon (IP) mode with quite low intensity near Dirac points corresponding to low doping level of AA-stacking graphite.

In the ΓA (out-plane) direction, the main peak starts at 0.26 eV, which originates from the collective intraband transitions. As *q* increases, the position of this peak increases and reaches its maximum 0.84 eV at the boundary of 1BZ with q∼0.85 Å${}^{-1}$, and then, it decreases. The intensity of the peak also increases firstly and then decreases as *q* increases, but it reaches its maximum at q∼0.1 Å${}^{-1}$.

### 3.3. Effect of Doping Level

**Figure 3:**
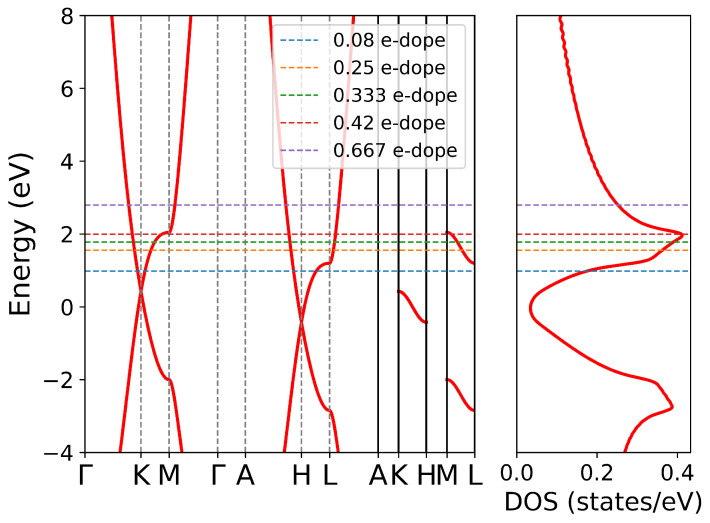
Band structure and Density of States (DOS) at different doping levels in rigid band approximation (RBA). The Fermi level at 0.08, 0.25, 0.333, 0.42, and 0.667 e-dope per unit cell are marked by light blue, orange, green, brown and violet dashed lines, corresponding to $E_{F}$ at 0.98, 1.555, 1.777, 1.992, and 2.793 eV, respectively.

**Figure 4:**
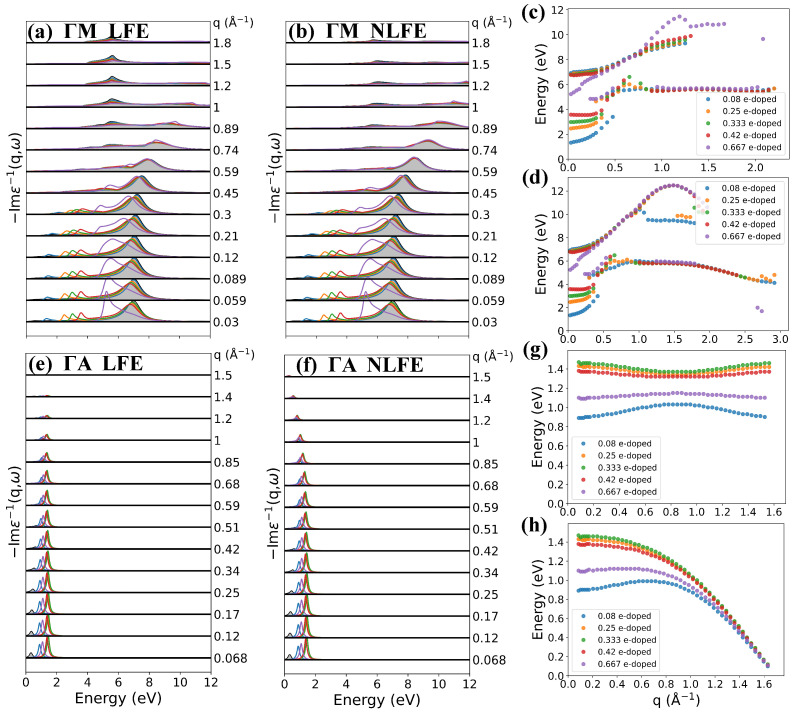
Calculated loss functions along the ΓM direction (**a**) with LFE, (**b**) without LFE and along the ΓA direction (**e**) with LFE, (**f**) without LFE at different doping levels (light blue line for 0.08 e-doped, orange line for 0.25 e-doped, green line for 0.333 e-doped, brown line for 0.42 e-doped and violet line for 0.667 e-doped per unit cell). (**c**,**d**,**g**,**h**) show positions of peaks of (**a**,**b**,**e**,**f**), respectively. The shaded background gives the loss functions of the no-doping case for comparison.

Intercalating different atoms in graphite will induce different doping levels, which is important to understand the behavior of electronic collective excitations. The induced electrons will occupy the $\pi^{*}$ band of carbon atoms, so the doping level can be regarded as an in-plane quantity to some extent. In order to study how this in-plane parameter influences the in-plane and out-plane collective excitation of electrons, we calculate the EELS of AA-stacked graphite at different doping levels. In this calculation, the doping effect is represented by ragid band approximation (RBA), which means only a rigid shift of the Fermi level, as shown in Figure 3. We calculated loss functions at different doping levels of 0.08, 0.25, 0.333, 0.42 and 0.667 e-doped per unit cell, correspoing to $3.75\times 10^{21}$, $1.17\times 10^{22}$, $1.56\times 10^{22}$, $1.97\times 10^{22}$, and $3.13\times 10^{22}$ cm${}^{-3}$ charge carrier density addition, respectively. The quite small difference between Figure 4a,b, especially at small *q*, illustrates that LFE is negligible in this direction. As the doping level increases at small *q*, both energies and intensities of IP mode increase, while π plasmon performs the inverse. When the doping level is larger than 0.42 e-doped per unit cell, the Fermi level will be lifted to the Van Hove singularity shown in Figure 3, and the π plasmon disappears. Because in this case, the $\pi^{*}$ band at M point is occupied and the corresponding interband transition is forbidden.

The dispersions of the in-plane modes with different electron doping levels are summarized in Figure 4c,d, corresponding to with and without inclusion of LFE, respectively. The π plasmon shows a parabolic-like positive dispersion with the increase of momentum transfer. As the doping level increases, at small *q*, more interband transitions are forbidden and the π plasmon energy shows a redshift; however, at large *q*, the forbidden effect is negligible and the π plasmon energy shows a blueshift. As a result, a higher doping level leads to a stronger parabolic-like positive dispersion of πP. The IP mode also shows a parabolic-like positive dispersion with the momentum transfer increase at low doping level. A higher doping level lifts the IP energy but reduces the dispersion, and even negetive dispersion appears at small *q* in 0.42 e-doped case. The negative dispersion of the in-plane IP mode has also been reported in CaC${}_{6}$[23] and SrC${}_{6}$ [24], which is explained by band structure effects.

In layered materials, plasmon properties are anisotropic and quite different between in-plane and out-plane direction. From Figure 4, it is obvious that LFE is crucial in ΓA direction, especially for the dispersion as shown in Figure 4g,h. When LFE is neglected, the energies of the out-plane IP mode decline to nearly 0 eV at the boundary of the second Brillouin zone (2BZ), while when LFE is taken into account, this IP mode shows a nearly flat band. The variation in energy is 0.14, 0.08, 0.09, 0.05 and 0.06 eV in 0.08, 0.25, 0.333, 0.42, and 0.667 e-doped per unit cell, respectively. This result agrees quite well with previous work in calculation for C${}_{6}$Li [24]. As the doping level lifts, the plasmon energy increases at first, reaches maximum around 0.333 e-doped per unit cell, and then drops down. The variation of intensities as a function of doping level shares the trend with the plasmon energies. Both density of states near the Fermi surface and the group velocities determine the IP energy. As shown in Figure 3, at low doping level, the density of state increases drastically with doping level increasing, and it reaches the top at 0.42 e-doped per unit cell. So, the IP energy firstly increases with doping level increase; then, it decreases due to the drop of density of state above Van Hove singlarity. However, the average group velocity along the ΓA direction of 0.42 e-dope is less than that of 0.333 e-dope. As a result, the IP energy of 0.42 e-dope is less than that of 0.333 e-dope.

### 3.4. Effect of Interlayer Distance and Hopping

Intercalating different atoms in layered materials will induce different interlayer distance and hopping, which are two important out-plane parameters to understand the behavior of electrons. In reality, they are always changed simultaneously, and it is hard to study their effect independently in experiments and first-principle calculations. In order to establish a simple picture how these two out-plane parameters influence the in-plane and out-plane electronic collective excitations, we tune them independently in our TB model and calculate the corresponding EELS.

**Figure 5:**
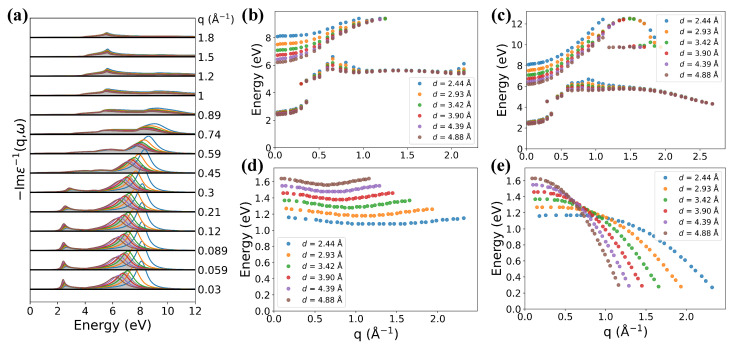
(**a**) Calculated loss functions along the ΓM direction with LFE at different interlayer distance (light blue line for d=2.44 Å, orange line for d=2.93 Å, green line for d=3.42 Å, brown line for d=3.90 Å, violet line for d=4.39 Å and brown line for d=4.88 Å). The shaded background gives the loss functions of d=3.70 Å case for comparison. (**b**,**c**) show the loss function peaks along the ΓM direction with and without LFE, respectively. (**d**,**e**) show the loss function peaks along the ΓA direction with and without LFE, respectively. The Fermi level is fixed at $E_{F}=1.555$ eV.

If we fix the interlayer hopping and tune the interlayer distance *d*, the band structrue has no change except for rescaling of reciprocal lattice vector along $c^{*}$-axis. So, the in-plane plasmon will be only tuned by the interlayer Coulomb correlation, while the out-plane plasmon can also be modified by rescaling effect along the $c^{*}$-axis. For example, as shown in Figure 5, the Fermi level $E_{F}$ is fixed at 1.555 eV, and the interlayer hopping $t_{⊥}$ is fixed at 0.21 eV. When *d* increases, the interlayer Coulomb interaction becomes weaker, so the in-plane IP and π plasmon energy decrease (Figure 5a–c). The intensity of IP increases as *d* increases, while that of π plasmon decreases. Figure 5b,c show the peak positions of the loss spectra along the ΓM direction with and without LFE, respectively. With the increase of *d*, both energies of IP and π plasmon decrease, but with different rates obviously. When *d* increases from 2.44 to 4.88 Å, the π plasmon energy decreases from 8.1 to 6.2 eV at small *q*, while the IP energy decreases only from 2.6 to 2.4 eV. As *q* increases, both IP and π plasmon show parabolic-like positive dispersion at different interlayer distances. With the interlayer distance decreasing, dispersions of IP mode remain unchanged with and without the inclusion of LFE, and we find a weaker dispersive feature of π plasmon mode with the inclusion of LFE. In addition, we plot the peak positions of the loss spectra along the ΓA direction in Figure 5d,e, where the out-plane IP energies indeed increase as *d* increases due to the $c^{*}$-axis rescaling effect.

**Figure 6:**
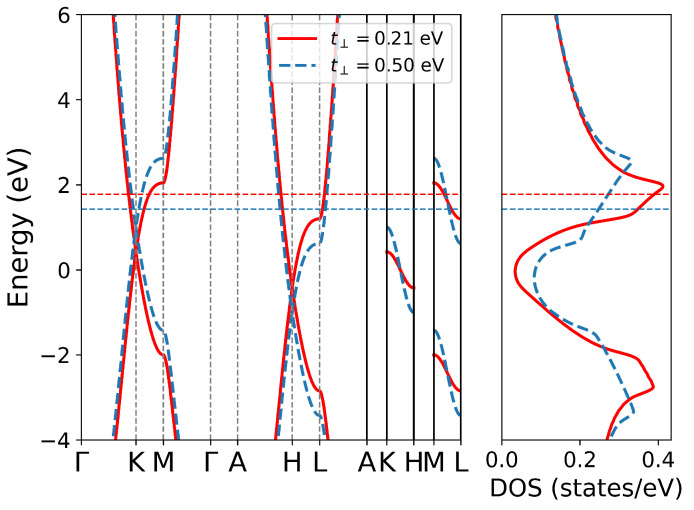
Band structure and density of states (DOS) at different interlayer hopping (red line for $t_{⊥}$ = 0.21 eV with $E_{F}$ = 1.555 eV and blue dashed line for $t_{⊥}$ = 0.50 eV with $E_{F}$ = 1.428 eV). The interlayer distance is fixed at 3.70 Å and the carrier density is fixed at $1.17\times 10^{22}$ cm${}^{-3}$.

**Figure 7:**
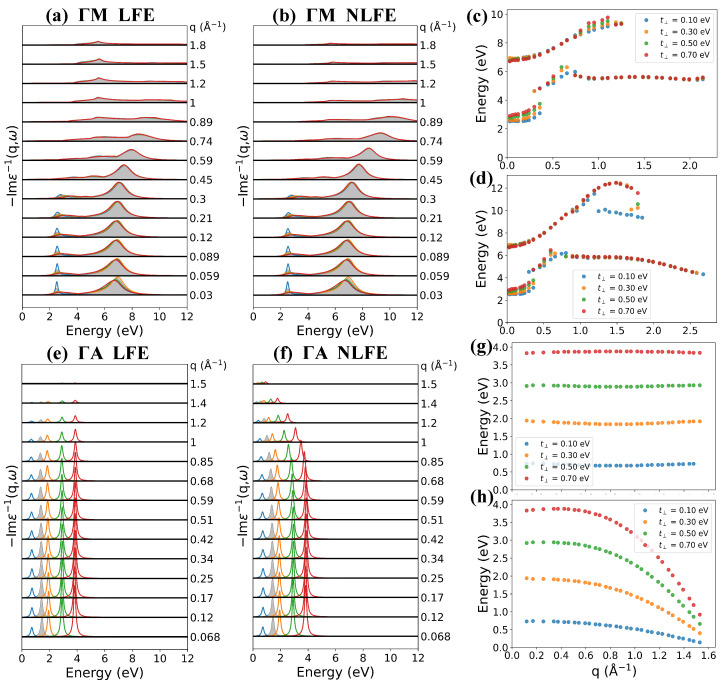
Calculated loss functions along the ΓM direction (**a**) with LFE, (**b**) without LFE and along the ΓA direction (**e**) with LFE, (**f**) without LFE at different interlayer hopping (light blue line for $t_{⊥}=0.10$ eV, orange line for $t_{⊥}=0.30$ eV, green line for $t_{⊥}=0.50$ eV and brown line for $t_{⊥}=0.70$ eV). (**c**,**d**,**g**,**h**) show positions of peaks of (**a**,**b**,**e**,**f**), respectively. The shaded background gives the loss functions of the no doping case for comparison.

As shown in Figure 6, larger interlayer hopping results in lower Fermi level and a larger energy difference between the different points along the $c^{*}$-axis, creating a larger group velocity along the out-plane direction. As a result, both the energy and weight of the out-plane IP mode increase dramatically with the increase of interlayer hopping $t_{⊥}$, as shown in Figure 7e–h. The effect of changing interlayer hopping on the band structure is similar to tuning the doping level at different $k_{⊥}$ plane seperately. For example, the energy difference between the ΓMK plane and AHL plane is $4t_{⊥}$, as shown in Table 2. Therefore, its effect on plasmons can be attributed to a combination of different doping level in all $k_{⊥}$ planes in 1BZ. Figure 7a–d shows the loss function along the ΓM direction (in-plane). The increase of $t_{⊥}$ has little effect on the in-plane π plasmon mode. The energy of the in-plane IP mode increases as $t_{⊥}$ increases, while the weight decreases significantly.

### 3.5. Effect of Stacking Order

In all known GICs, the stacking order of graphene layer is AAA [1], which is different from the AB or ABC stacking order in natural graphite. The stacking order can modify the interlayer coupling and electronic structure consequently. For example, in small-rotation-angle twisted bilayer graphene, the moiré superlattice alters the electronic properties significantly and has led to observations of exotic emergent electronic properties such as superconductivity and strong correlated states [35,36].

**Figure 8:**
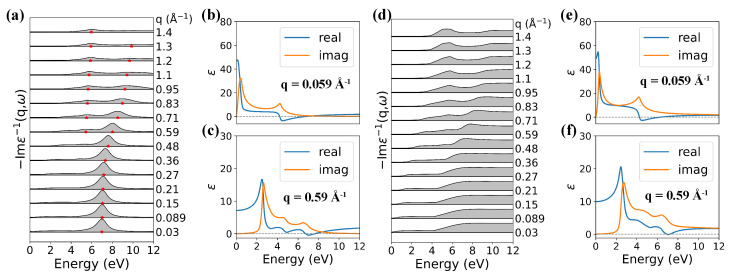
Calculated loss functions along the ΓM direction of AB (**a**) and ABC (**d**) graphite with LFE. The peak positions are marked by red stars. Real and imaginary part of dielectric function at (**b**) q=0.059 Å${}^{-1}$ and (**c**) q=0.59 Å${}^{-1}$ for AB graphite and (**e**) q=0.059 Å${}^{-1}$ and (**f**) q=0.59 Å${}^{-1}$ for ABC graphite.

The plasmon properties of AB and ABC stacking graphite are studied based on our TB model (for more details, please see Appendix D). The loss function of AB graphite along the ΓM direction is almost the same as that of AA graphite. The main feature of the loss spectra is a strong π plasmon peak around 7 eV at small *q*, and the π plasmon shows positive-parabolic dispersion with an increase of *q*, which agrees very well with previous work [33], as shown in Figure 8a. The real and imaginary parts of dielectric functions at q=0.059 Å${}^{-1}$ in Figure 8b and at q=0.59 Å${}^{-1}$ in Figure 8c are similar with that of AA graphite, except that no zero point of Re[ϵ] appears at E<4 eV due to the lack of charge carriers. However, no plasmon peaks are observed in ABC graphite in the low-energy region (0–12 eV), as shown in Figure 8d. The collective character in the loss function is cofirmed by the real and imaginary parts of the dielectric function at q=0.059 Å${}^{-1}$ in Figure 8e and q=0.59 Å${}^{-1}$ in Figure 8f. We can see that at energy around 7 eV, Re[ϵ] crosses the zero line with a positive derivative at q=0.059 Å${}^{-1};$ however, Im[ϵ] is still too large, implying a strong Landau damping effect. As a result, π plasmon will have a very large linewidth, and no peak appears in the loss function. Along the out-plane ΓA direction, no prominent plasmon peaks appear in the low-energy region (0–12 eV) for both AB and ABC stacking graphite due to the low level of charge carrier concentration.

As the doping level increases, the density of state and the group velocity near the Fermi surface change rapidly according to the band structure, and more interband transitions from π band to $\pi^{*}$ band are forbidden at small *q*. As a result, with the increase of doping level, the energy blueshifts for in-plane IP mode, blueshifts at first and then redshifts for out-plane IP mode, and blueshifts at small *q* but redshifts at large *q* for in-plane π plasmon mode. The variation in the intensity of plasmon peaks is the same as plasmon energies. When the doping level is so large that the $\pi^{*}$ band at the M point is occupied, the in-plane π plasmon disappears as a result of a forbidden interband transition from the π band to $\pi^{*}$ band.

With the interlayer distance increasing, the band structrue has no change except for rescaling of the reciprocal lattice vector along the $c^{*}$-axis when the interlayer hopping remains unchanged, and the interlayer Coulomb correlation becomes weaker. As a consequence, the energy redshifts slightly for the in-plane IP mode, redshifts remarkably for the in-plane π plasmon mode, and blueshifts for the out-plane IP mode. The variation in the strength of plasmon peaks is the same as plasmon energies for the in-plane π plasmon and out-plane IP modes, but it is opposite for the in-plane IP mode. The effect of changing interlayer hopping on the band structure is similar to tuning the doping level at different $k_{⊥}$ planes separately and group velocity along the out-plane direction. We find that the interlayer hopping has nearly no effect on in-plane π plasmon mode, but it affects the IP mode dramatically. As the interlayer hopping increases, the energies of both the in-plane and out-plane IP mode blueshift, and the strength of plasmon peak increases for the out-plane mode but decreases for the in-plane mode. AB-stacking graphite shows few differences with AA-graphite, while no plasmon peaks appear in ABC-stacking graphite because of the strong Landau damping effect.

A comparison of the excitation spectra obtained with and without inclusion of the local-field effects demonstrates that in layered AA stacked graphite, the LFE have a significant impact on the dielectric properties, especially along the out-plane direction, where they flatten the out-plane IP dispersion at least in the second Brillouin zone (2BZ).

## 4. Conclusions

Based on the computing resource and precision limitation of current simulation on layered materials, we have constructed a TB model of AA-stacking graphite to mimic GICs and investigated the plasmons properties within the RPA framework, with high efficiency and sufficient precision. The local-field effects are involved in our model; the effects of doping level, interlayer distance, interlayer hopping, and stacking order on 2D π plasmon and 3D intraband plasmon modes are studied independently; and the corresponding evolutions of plasmons are presented clearly. Our results are in very good agreement with the previous experimental and calculation work. This tight-binding calculation does not need a self-consistent process and the basis set contains only several orbitals, so it is very computationally efficient. At the same time, the precision is higher than many methods and satisfies most of the prediction requirements. Our method is easy to extend to other intercalation materials with unlimited intercalation species and has great significance for understanding how plasmons are tuned in layered materials.   

## Figures and Tables

**Table 1:** Hopping parameters $t_{i}$ (in eV) assigned to the simple TB Hamiltonian of AA-stacking graphite. *d* is the distance between the atomic sites on which the interacting orbitals are centered.

*i*	$t_{i}$ (eV)	*d* (Å)
1	−3.24	1.41
2	0.36	2.44
3	−0.41	2.82
4	0.095	4.23
⊥	0.21	3.7

**Table 2:** The analytical energy eigenvalues at high-symmetry points.

Points	Energy Eigenvalues
Γ	$\epsilon_{p}+2t_{⊥}+6t_{2}+6t_{4}\pm 3\left(t_{1}+t_{3}\right)$
K	$\epsilon_{p}+2t_{⊥}$
M	$\epsilon_{p}+2t_{⊥}-4t_{2}-2t_{4}\pm \frac{\sqrt{3}}{2}\left(t_{1}-3t_{3}\right)$
A	$\epsilon_{p}-2t_{⊥}+6t_{2}+6t_{4}\pm 3\left(t_{1}+t_{3}\right)$
H	$\epsilon_{p}-2t_{⊥}$
L	$\epsilon_{p}-2t_{⊥}-4t_{2}-2t_{4}\pm \frac{\sqrt{3}}{2}\left(t_{1}-3t_{3}\right)$

**Figure A1:**
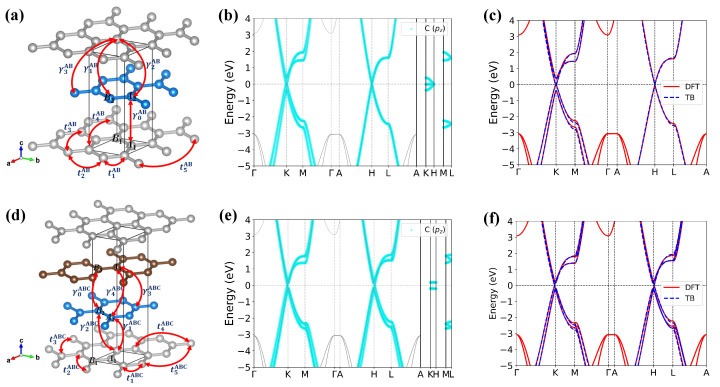
Lattice and electronic structure of AB and ABC graphite. (**a**,**d**) are the lattice structure of AB and ABC graphite, respectively. Carbon atoms in different layers are labeled in different colors (gray and blue in AB case, and gray, blue and brown in ABC case). The red arrows denote the interatomic tight-binding hopping parameters. (**b**,**e**) are the electronic band structure of AB and ABC graphite, respectively, with the $p_{z}$ orbitals of C atoms in cyan. (**c**,**f**) are the comparison of band structure (DFT in red line and TB in blue dashed line) for AB graphite and ABC graphite, respectively.

## Data Availability

Not applicable.

## Appendix A. Tight-Binding Model

We constuct the Bloch-like basis functions in Convention I [37] as

(A1) $$\begin{array}{c} |\alpha ,k\rangle =\frac{1}{\sqrt{N}}\sum_{L}e^{ik\cdot (R_{L}+\tau_{\alpha })}|\alpha ,L\rangle , \end{array}$$

where *N* is the number of unit cells, $R_{L}$ is the lattice vector, $\tau_{\alpha }$ is the position vector of α orbital within the unit cell, and |α,L〉 is the tight-binding orbital. The Hamiltonian matrix expressed in the basis is

(A2) $$\begin{array}{c} H_{\alpha \alpha^{\prime }}\left(k\right)=\langle \alpha^{\prime },k|H|\alpha ,k\rangle =\sum_{R}e^{ik\cdot (R+\tau_{\alpha }-\tau_{\alpha^{\prime }})}H_{\alpha \alpha^{\prime }}\left(R\right), \end{array}$$

where

(A3) $$\begin{array}{c} H_{\alpha \alpha^{\prime }}\left(R\right)=\langle \alpha ,0|\hat{H}|\alpha^{\prime },R\rangle . \end{array}$$

The energy eigenvalues and Bloch eigenstates can be obtained by diagonalization of the Hamiltonian matrix and the Bloch eigenstates are then expanded as

(A4) $$\begin{array}{c} |nk\rangle =\sum_{\alpha }C_{\alpha }\left(nk\right)|\alpha ,k\rangle . \end{array}$$

## Appendix B. Approximations in Practical

Assuming that the localized basis sets are orthonormal and localized enough, the charge-density wave becomes

(A5) $$\begin{array}{c} A_{s}\left(q,G\right)=\langle \nu ,0|e^{-i(q+G)\cdot r}e^{iq\cdot \tau_{\mu }}|\mu ,L\rangle \delta_{\nu \mu }\delta_{L0}, \end{array}$$

furthermore, we have

(A6) $$\begin{array}{c} A_{s}\left(q,G=0\right)\approx 1. \end{array}$$

The $N_{ss^{\prime }}\left(q,\omega \right)$ becomes

(A7) $$\begin{array}{cc} N_{ss^{\prime }}\left(q,\omega \right)\approx & \frac{2}{N}\sum_{nn^{\prime }k}\frac{f_{nk}-f_{n^{\prime }k+q}}{\hbar \omega +E_{nk}-E_{n^{\prime }k+q}+i\hbar \eta }\\ \; & \times C_{\mu }^{*}\left(nk\right)C_{\mu }\left(n^{\prime }k+q\right)C_{\mu^{\prime }}^{*}\left(n^{\prime }k+q\right)C_{\mu^{\prime }}\left(nk\right), \end{array}$$

the Coulomb interaction (9) between the charge-density waves becomes

(A8) $$\begin{array}{c} V_{\alpha \alpha^{\prime }}\left(q\right)\approx \sum_{m}{\;}^{\prime }\frac{e^{-iq\cdot (R_{m}+\tau_{\mu }-\tau_{\mu^{\prime }})}}{|R_{m}+\tau_{\mu }-\tau_{\mu^{\prime }}|} \end{array}$$

where the prime indicates that the sum excludes the intra-atomic term. In practical terms, we obtain the intra-atomic Coulomb repulsion from Harrison’s fitting [38].

We usually constract the TB model only using orbitals which are close to the Fermi level, so an effective dielectric constant $\epsilon_{0}$, which includes a high-energy screening process, has to be induced. The dielectric function matrix becomes

(A9) $$\begin{array}{cc} \epsilon_{GG^{\prime }}\left(q,\omega \right)= & \epsilon_{0}\delta_{GG^{\prime }}-v\left(q+G\right)\\ \; & \times \sum_{ss^{\prime }}A_{s}\left(q,G\right)N_{ss^{\prime }}\left(q,\omega \right)A_{s^{\prime }}^{*}\left(q,G^{\prime }\right), \end{array}$$

and the inverse dielectric matrix

(A10) $$\begin{array}{cc} \epsilon_{GG^{\prime }}^{-1}\left(q,\omega \right)=\frac{\delta_{GG^{\prime }}}{\epsilon_{0}} & +\frac{v(q+G)}{\epsilon_{0}}\\ \; & \times \sum_{ss^{\prime }}A_{s}\left(q,G\right)S_{ss^{\prime }}\left(q,\omega \right)A_{s^{\prime }}^{*}\left(q,G^{\prime }\right), \end{array}$$

where

(A11) $$\begin{array}{cc} S_{ss^{\prime }}\left(q,\omega \right)= & \sum_{s_{1}}N_{ss_{1}}\left(q,\omega \right)T_{s_{1}s^{\prime }}^{-1},\\ T_{\alpha \alpha^{\prime }}\left(q,\omega \right)= & \epsilon_{0}\delta_{\alpha \alpha^{\prime }}-\sum_{\alpha_{1}}V_{\alpha \alpha_{1}}N_{\alpha_{1}\alpha^{\prime }}\left(q,\omega \right) \end{array}$$

## Appendix C. First-Principle Calculation Details

In order to obtain a reliable TB model, the geometrical optimization and electronic properties are performed by first-principle calculations. We fixed the interlayer distance at 3.7 Å for all stacking order, corresponding to Li-intercalated graphite [11], and relaxed the atomic structures according to the force and stress performed by density functional theory (DFT) using the Quantum Espresso (QE) [39,40]. The norm-conserving pseudopotentials [41,42] and local density approximation (LDA) [43,44] exchange-correlation functional were adopted. The cutoff energy was set to 100 Ry after convergence tests. We used gamma-centered 16×16×10, 16×16×5 and 16×16×4 **k**-mesh for AA, AB and ABC stacking order respectively, and a Methfessel–Paxton [45] smearing width of 0.01 Ry in the self-consistent calculations. The lattice parameters and atomic positions were fully relaxed until the remanent forces are less than $1\times 10^{-4}$ Ry/Bohr.

## Appendix D. Tight-Binding Model of AB and ABC Stacking Graphite

AB graphite has four atoms in the unit cell and four π band near the Fermi level, as shown in Figure A1a,b. The conduction band and valence band match at the Fermi level at K and H points, which shows semi-metal property. As a result, the carrier concentration in AB graphite is much lower than that in AA graphite. In order to establish a reliable TB model, we employ five intralayer parameters and four interlayer parameters, as shown in Figure A1a and Table A1. The on-site energies of C atoms $E_{0}$ in the same layer have a difference Δ due to the crystal field effect. In order to make a comparison between different stacking orders, we utilize a hexgonal unit cell of rhombohedral or ABC graphite, which contains six atoms and six π bands near the Fermi level, as shown in Figure A1d,e. At K and H points, a small gap is opened, and the conduction band and valence band intersect at the Fermi level near the K and H points. We employ five intralayer parameters and five interlayer parameters, as shown in Figure A1d and Table A1. The electronic band structures derived from our TB model matches very well with the DFT calculation, as shown in Figure A1c for AB graphite and (f) for ABC graphite, implying the reliability of our TB model.

**Table A1:** TB parameters for AB stacking and ABC stacking graphite. All values are in eV. The TB Hamiltonian is valid in the whole three-dimensional Brillouin zone of graphite.

Stacking Order	AB	ABC
$t_{1}$	−3.04	−3.042
$t_{2}$	0.254	0.267
$t_{3}$	−0.312	−0.313
$t_{4}$	0.06	0.066
$t_{5}$	−0.022	−0.018
$\gamma_{0}$	0.177	0.177
$\gamma_{1}$	0.098	0.048
$\gamma_{2}$	0.045	−0.042
$\gamma_{3}$	−0.035	0.084
$\gamma_{4}$		−0.024
$E_{0}$	0.317	0.333
Δ	0.004	0
